# Editorial: Physiology of human myopathies

**DOI:** 10.3389/fphys.2025.1597211

**Published:** 2025-04-07

**Authors:** Aldrin V. Gomes, Jose R. Pinto, Danuta Szczesna-Cordary

**Affiliations:** ^1^ Department of Neurobiology, Physiology, and Behavior, University of California, Davis, Davis, CA, United States; ^2^ Department of Biomedical Sciences, Florida State University College of Medicine, Tallahassee, FL, United States; ^3^ Department of Molecular and Cellular Pharmacology, University of Miami, Miami, FL, United States

**Keywords:** cardiac muscle, cardiomyopathy, nutraceuticals, tropomyosin, troponin, skeletal muscle atrophy, Hg exposure

Cardiac and skeletal muscle disorders often result from molecular irregularities that affect muscle function, contraction, and protein degradation processes. The research studies and reviews presented in this Research Topic provide valuable insights into the molecular mechanisms underlying cardiomyopathy and skeletal muscle disorder, as well as the toxic effects of heavy metal exposure to the heart. These studies also explore potential therapeutic interventions to mitigate the consequences of human myopathies. We summarize and discuss findings from these experimental studies and reviews, offering insights into these interconnected topics.

The study by Yang et al. addresses the uncoupling between PKA-mediated phosphorylation of troponin I and muscle regulation due to cardiomyopathy-linked mutations in sarcomeric actin (*ACTC* gene) and troponin T (*TNNT2* gene) (Marston and Pinto, 2022). It also explores the ability of small molecules to restore coupling and normal heart function. Specifically, the authors investigated the effects of nutraceutical compounds - silybin B, resveratrol, and epigallocatechin-3-gallate (EGCG) - on restoring the heart’s ability to relax during diastole (lusitropy) in two models of cardiomyopathy (Yang et al.). These nutraceuticals were found to interact with cardiac troponin, restore the PKA-mediated modulation of myofilament Ca^2+^-sensitivity, thus improving diastolic function in hearts containing the *ACTC*-E99K and *TNNT2*-R92Q mutations. A strength of this study is its use of complementary experimental approaches, including *in vitro* (cell-based), *in vivo* (animal), and *in silico* (computational modeling) techniques. Molecular dynamics simulations revealed that silybin B, EGCG, and resveratrol restored the phosphorylation-induced change in troponin C helix A/B angle and interdomain angle to the levels observed in wild-type conditions in the presence of the *TNNC1*-G159D mutation. Previous studies have shown that resveratrol reduces oxidative stress, while EGCG has anti-inflammatory properties (Payne et al., 2023; Wei et al., 2023). However, the discovery by Yang et al. that these nutraceutical compounds restore lusitropy by interacting with troponin adds a novel dimension to their therapeutic potential. Recent clinical trials using nutraceuticals such as L-carnitine, hawthorn and n-3 PUFA have shown improvements in functional parameters and quality of life in heart failure patients (Cicero et al., 2020). Further research on these compounds and their potential benefits for cardiac function may lead to novel treatment options for cardiomyopathy.

The article by Creso et al. investigated the molecular mechanisms underlying hypocontractility caused by the M8R tropomyosin (*TPM1* gene) mutation, which has been associated with dilated cardiomyopathy in humans. Using both *in silico* (computational) and *in vitro* (experimental) models, the authors revealed that the *TPM1*-M8R mutation disrupts normal interactions between tropomyosin and actin-myosin filaments, leading to reduced cardiac muscle contraction efficiency (Creso et al.). Previous studies have shown that the M8R mutation, located at the overlap junction of tropomyosin strands, decreases Ca^2+^-sensitivity and thin filament cooperativity, and weakens tropomyosin’s ability to bind actin, perturbing thin filament regulatory function (Racca et al., 2020). In this study, the authors extended these results using a human tissue engineering approach as well as *in silico* modeling, circular dichroism (CD), actin co-sedimentation, and *in vitro* gliding filament assays to provide mechanistic insight into how the mutation-induced molecular effects influence more physiologically relevant cardiac tissue function. The development of more efficient and cost-effective gene modification techniques, as presented in (Creso et al.), suggests that these approaches may become useful therapeutic options for patients with severe cardiomyopathy caused by mutations in sarcomeric proteins.

Pang et al. reviewed the role of the ubiquitin-proteasome pathway (UPP) in skeletal muscle atrophy, a condition characterized by the loss or deterioration of muscle mass and tissue due to an imbalance between protein synthesis and degradation. The UPP is a highly complex pathway present in all tissues, involving hundreds of different proteins that work together to remove damaged or unwanted proteins (Gilda and Gomes 2017). It functions by tagging target proteins with ubiquitin, marking them for degradation by the proteasome. Proteomic and other studies of various conditions, including muscle disuse, malnutrition, aging, and neuromuscular diseases, have shown that many components of the UPP are altered in these diseases. The review highlights MuRF1 and Atrogin-1, two highly abundant E3 ubiquitin ligases in muscle that are known to contribute to progressive muscle atrophy and weakness following their upregulation. These ligases are directly involved in muscle atrophy by degrading muscle proteins such as actin and myosin heavy chains. The available techniques and protocols for current interventions to prevent or reverse muscle atrophy, including resistance training (Hu et al., 2024), are not well-defined or well-described. A previous review of muscle atrophy suggests that no single treatment is sufficient for patients (Huang et al., 2022). The review by Pang et al. urges scientists to investigate the role of the UPS in muscle atrophy and its underlying mechanisms to develop new treatment strategies.

Finally, the study by Bello et al. investigated the effects of chronic mercury (Hg) exposure on arrhythmia and mortality following an acute myocardial infarction (MI) in rats (Bello et al.). Prolonged Hg exposure can occur through food, occupational work, environmental sources, and household products. The authors found that Hg exposure has exacerbated the severity of the adverse effects of MI in the MI-Hg groups of rats compared to the MI-only groups of rats, leading to increased mortality rates. Hg has toxic effects on the heart but their mechanisms are only partially understood and are thought to involve ion channel dysfunction, oxidative stress, and defects in heart contractility (Furieri et al., 2011; Guallar et al., 2002). The findings by Bello et al. are in line with previous studies that have established the cardiovascular toxicity of environmental Hg exposure. They highlight the importance of public health initiatives to prevent human exposure to environmental Hg, as Hg should be recognized as a risk factor that worsens cardiac ischemic injury and increases the mortality rate among patients with acute MI.

In conclusion, the articles of this Research Topic enhanced our understanding of diverse yet interrelated aspects of cardiovascular and muscular health, including cardiomyopathy, skeletal muscle atrophy, and toxic exposure to environmental Hg. The mentioned interconnections of the pathways suggest that future research and treatment should integrate the knowledge from different disease mechanisms in order to develop better treatment strategies. Ultimately, gaining deeper insight into the interplay of these mechanisms will be essential for advancing clinical interventions and personalized medicine.
